# Incomplete DRB4-dependence of the DCL4-mediated antiviral defense

**DOI:** 10.1038/srep39244

**Published:** 2016-12-16

**Authors:** Xiaofeng Zhang, Xiuchun Zhang, Kunxin Wu, Zhixin Liu, Dawei Li, Feng Qu

**Affiliations:** 1 Institute of Tropical Bioscience and Biotechnology, Chinese Academy of Tropical Agricultural Sciences/ Key Laboratory of Biology and Genetic Resources of Tropical Crops, Ministry of Agriculture, P.R. China; 2 Department of Plant Pathology, The Ohio State University, Wooster, Ohio, USA; 3 Fujian Province Key Laboratory of Plant Virology/ Institute of Plant Virology, Fujian Agriculture and Forestry University, Fuzhou, Fujian, P.R. China; 4 State Key Laboratory of Agro-Biotechnology, College of Biological Sciences, China Agricultural University, China

## Abstract

The double-stranded RNA-binding protein DRB4 of Arabidopsis was shown previously to contribute to the DICER-LIKE 4 (DCL4)-mediated biogenesis of viral small interfering RNAs (vsiRNAs) of 21 nucleotides (nt) in size. However, it is unclear whether all 21-nt vsiRNAs are dependent on this DRB4-DCL4 partnership. To resolve this question, we generated *dcl2drb4* and *dcl4drb4* double knockout mutants, and subjected them to infections with CPB-CC-PDS, a turnip crinkle virus mutant capable of inducing silencing of the *PHYTOENE DESATURASE* gene. The *dcl2drb4* double knockouts caused a far smaller loss of antiviral silencing than *dcl2dcl4.* In addition, although both *drb4* and *dcl4* single mutants permitted a consistent (but small) increase in viral RNA levels, the *drb4* mutant correlated with a less pronounced reduction of 21-nt vsiRNAs. Therefore, a substantial subset of DCL4 antiviral activity is DRB4-independent, and may involve other DRB proteins that compensate for loss of DRB4.

Antiviral RNA silencing in plants enlists DICER-LIKE 2 and 4 (DCL2 and 4), and DCL3 to a lesser extent, to process double-stranded RNA (dsRNA) of virus origin into viral small interfering RNAs (vsiRNAs) of 21–24 nucleotides (nt) in size^1^,^2^,^3^. It was further established that DCL-mediated processing of dsRNA requires the participation of members of the dsRNA-binding protein (DRB) family^4^,^5^. For example, processing of endogenous precursor of microRNAs (pre-miRNAs) by DCL1 requires HYL1 (DRB1), whereas processing of the trans-acting siRNA (tasiRNA) precursors by DCL4 requires DRB4^6^,^7^. More recently it was reported that processing of geminiviral dsRNA by DCL3 requires DRB3^8^. However, the DCL-DRB relationship may not be as specific as initially thought^4^, as DCL4 processing of tasiRNA precursors other than the tasiRNA3 precursor appears to be less dependent on DRB4^7^. This suggests that either DCL4 could process certain type of dsRNA independent of a DRB, or it also collaborates with other DRBs.

Among the five DRBs encoded by the model plant Arabidopsis, the role of HYL1/DRB1 in DCL1-mediated miRNA biogenesis is best understood^6^,^9^,^10^. On the other hand, DRB4 and DRB3 are the only DRBs found to participate in antiviral silencing^2^,^5^,^8^,^11^,^12^. In addition to participating in the biogenesis of vsiRNAs directly, DRB4 has also been shown to play a role in perturbing viral RNA translation^13^. However, it is important to note that disruption of DRB4 did not lead to a complete loss of 21-nt vsiRNAs^2^. This suggests that, similar to certain endogenous tasiRNA precursors, some viral dsRNA might also bypass the need for DRB4.

In this report, we attempted to clarify what extent the antiviral function of DCL4 depends on DRB4. To this end, we generated *Arabidopsis dcl2drb4* and *dcl4drb4* double knockout mutants, and subjected them, along with *drb4, dcl2, dcl4* single mutants, and *dcl2dcl4* double mutants, to infections with CPB-CC-PDS, a turnip crinkle virus mutant capable of inducing silencing of the *PHYTOENE DESATURASE* gene. Our results showed that the *dcl2drb4* double knockouts caused a far smaller loss of antiviral silencing than *dcl2dcl4.* In addition, although both *drb4* and *dcl4* single mutants permitted a consistent (but small) increase in viral RNA levels, the *drb4* mutant correlated with a less pronounced reduction of 21-nt vsiRNAs. Therefore, a substantial subset of DCL4 antiviral activity is DRB4-independent, and may involve other DRB proteins that compensate for loss of DRB4.

## Results and Discussion

We and others have previously determined that the *dcl2dcl4* double knockout mutant of Arabidopsis was nearly completely defective at antiviral silencing against RNA viruses^2^,^14^,^15^. Therefore, if DCL4 requires DRB4 for the entirety of its antiviral function, then the *dcl2drb4* and *dcl2dcl4* mutants would be predicted to respond to virus attacks in a similar manner. To test this prediction, we generated *dcl2drb4,* as well as *dcl4drb4* double knockouts by crossing *dcl2-1* and *dcl4-2* with *drb4-1*. Interestingly, both *dcl2drb4* and *dcl4drb4* mutants morphologically resembled *drb4* single mutant, without the size reduction observed by Nakazawa and colleagues^16^. Since we also did not observe the dramatic anthocyanin over-accumulation in *dcl4* or *drb4* single mutants reported by these authors^16^, we reason that the smaller size of their *dcl4drb4* plants might be caused by the specific growth conditions they used. The resulting homozygous double mutants, along with *drb4, dcl2, dcl4* single mutants, and *dcl2dcl4* double mutants, were subjected to infections with CPB-CC-PDS which is a turnip crinkle virus (TCV) mutant encoding an attenuated suppressor of RNA silencing contains an insertion of 90 nucleotides (nt), derived from the *PHYTOENE DESATURASE (PDS)* gene of Arabidopsis. The *PDS* insert induces modest PDS silencing in CPB-CC-PDS-infected plants, providing a visual indicator for the silencing-inducing capability of this viral mutant^16^. Wild type TCV was not used because its strong silencing suppressor obscures the functional manifestation of plant mutants^2^,^14^.

**Figure 1:**
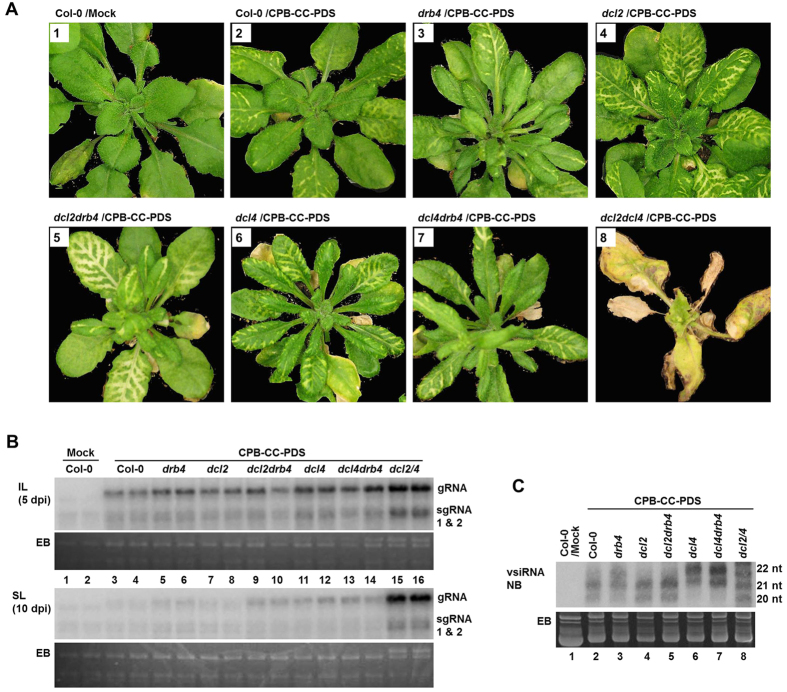
Loss of antiviral silencing activity in *dcl2drb4* double knockouts is substantially less debilitating than in *dcl2dcl4* mutant. (**A**) PDS silencing induced by CPB-CC-PDS infection of various Arabidopsis mutant plants. Images were recorded at 25 dpi. (**B**) Northern blot hybridization of the total RNAs extracted from the plants shown in A. Each RNA sample was extracted from six inoculated or systemic leaves (IL or SL) pooled from six plants. To further even out variabilities, two different RNA samples were analyzed per treatment. The probe was a mix of five ^32^P-labelled oligos complementary to TCV genomic RNA (gRNA). EB: ethidium bromide-stained Northern gel. sgRNA: subgenomic RNA. (**C**). Northern blot hybridization of vsiRNAs. See Qu *et al*.^2^ for a detailed procedure.

The CPB-CC-PDS-infected plants were reared in growth chambers with a constant temperature of 18 °C, and 14 hour daylight. As shown in Fig. 1A, plants photographed at 25 days post inoculation (dpi) showed varying degrees of PDS silencing along the secondary veins depending on the mutant background. It is worth noting that the extent of PDS silencing in *drb4* plants was almost indistinguishable from wildtype Col-0 plants (Fig. 1A, compare panels 2 and 3), despite a small but consistent increase in viral RNA levels in both inoculated and systemic leaves (IL and SL; Fig. 1B, compare lanes 5, 6 with 3, 4). In contrast, *dcl2* mutant plants showed substantially more intense PDS silencing than both *drb4* and Col-0 (Fig. 1A, compare panels 2, 3, and 4), despite a much smaller increase in viral RNA levels (Fig. 1B, IL and SL, compare lanes 7, 8 with 5, 6). Therefore, unlike in *dcl2*, increased viral RNA accumulation in *drb4* did not result in enhanced target silencing. This suggests that in addition to contributing to DCL4-mediated dsRNA processing, DRB4 might also be needed downstream for vsiRNA-guided slicing of complementary mRNAs.

We also examined the vsiRNA profiles in these plants. In agreement with our previous studies^15^, the CPB-CC-PDS-infected Col-0 plants accumulated predominantly DCL4-dependent 21-nt siRNAs accompanied by a barely detectable 22-nt band (Fig. 1C, lane 2). The 20-nt band near the bottom of lanes 2, 4, and 5 likely have derived from 21-nt vsiRNAs, as it was absent in *dcl4* and *dcl4drb4* plants (lane 6 and 7). In *drb4*, both the 20-nt and 21-nt vsiRNA bands were weakened, accompanied by a visible enhancement of the 22-nt band. This suggests that DRB4 does act similarly as DCL4 in that its disruption led to reduction of 21-nt vsiRNAs and increase of 22-nt vsiRNAs. However, in *drb4* plants the reduction of 21-nt vsiRNAs and its 20-nt derivative was much less dramatic than in *dcl4* plants, and the corresponding increase of 22-nt vsiRNAs was also much less pronounced. Therefore, DRB4 is needed for the biogenesis of some, but not all, vsiRNAs produced by DCL4. Conversely, DCL4 is capable of processing certain types of viral dsRNAs into 21-nt vsiRNAs independently of DRB4.

Further strong support for the existence of a DRB4-independent subset of DCL4 activity came from examining *dcl2drb4* mutant plants. As shown in Fig. 1A, panel 5, the CPB-CC-PDS-infected *dcl2drb4* showed a PDS silencing pattern that is slightly more intense than *dcl2* single mutant, as evidenced by slightly wider and more intense bleaching of secondary veins. More tellingly, the viral RNA levels in these plants were also only slightly increased compared with *drb4* or *dcl2* single mutants (Fig. 1B, IL and SL, lanes 9 and 10). All these findings are in stark contrast with the *dcl2dcl4* mutants, where high levels of CPB-CC-PDS RNA accumulation caused much more severe viral symptoms that eventually led to the death of many plants, but with no PDS silencing^15^ (also see Fig. 1A, panel 8, and Fig. 1B, lanes 15 and 16). Therefore, DCL4, or at least a substantial fraction of it, was still functional in the *dcl2drb4* mutant, and acted potently to destruct viral RNA. Indeed, there was actually a modest increase of 21-nt vsiRNA levels in the *dcl2drb4* mutant than in *dcl2* single mutant (Fig. 1C, lane 4 and 5), probably reflecting the small increase in the level of viral RNA that could serve as templates for DCL4.

Finally, consistent with a potential role of DRB4 in a downstream step^13^, PDS silencing was actually less intense in *dcl4drb4* double mutant than in *dcl4* single mutant (Fig. 1A, panels 6 and 7), despite similar levels of viral RNA in the two kinds of plants (Fig. 1B, lanes 11–14), as well as similar patterns of vsiRNAs (Fig. 1C, lanes 6 and 7). Together our results confirmed a critical role of DRB4 in DCL4-mediated 21-nt vsiRNA biogenesis and antiviral defense, as evidenced by a modest increase in viral RNA levels, and a detectable decline of 21- and 22-nt vsiRNA ratio in virus-infected *drb4* plants. These changes are similar to, but less penetrating than, in *dcl4* plants, suggesting that DRB4 and DCL4 participate in the same vsiRNA-producing pathway. Surprisingly however, DCL4 could accomplish most of its antiviral function in the absence of DRB4, as knocking out DRB4 in *dcl2* background (as in *dcl2drb4*) had a far smaller effect than knocking out DCL4 itself (as in *dcl2dcl4*) on viral RNA accumulation, target silencing, and disease symptoms. How do we explain these findings? We reason that while DRB4 clearly has a unique role in the biogenesis of a subset of 21-nt vsiRNAs, the function of these vsiRNAs could be readily be compensated by other 21-nt vsiRNAs produced without the involvement of DRB4. Although it is possible that DCL4 could process certain types of viral dsRNAs without any DRBs, it appears to be more plausible that one or more other members of the five-member DRB family could substitute for the loss of DRB4 and assist DCL4 in 21-nt vsiRNA biogenesis. Our next goal will be identify these additional DRBs that contribute to antiviral silencing against RNA viruses.

## Materials and Methods

### Construct

CPB-CC-PDS, has been described in a previous study^15^. Briefly, it differs from wildtype TCV at two positions: (i) it contains an arginine (R) to threonine (T) mutation at position no. 130 of TCV capsid protein (CP) that substantially compromised the ability of TCV CP to suppress RNA silencing^14^; (ii) it additionally contains an insertion of 90 nucleotides (nt), derived from the *PHYTOENE DESATURASE (PDS)* gene of Arabidopsis. The *PDS* insert, located immediately downstream of the CP coding region, induces modest PDS silencing in CPB-CC-PDS-infected plants, providing a visual indicator for the silencing-inducing capability of this viral mutant^15^.

### Plant materials

The sources of Col-0, *dcl, drb4* mutant plants have been described previously^2^. *dcl2drb4,* as well as *dcl4drb4* double knockouts were generated by crossing *dcl2-1* and *dcl4-2* with *drb4-1* respectively. Uninfected Arabidopsis plants were reared in a growth room set at 20 °C, with 14 h of daylight. After inoculation with *in vitro* transcripts of TCV derivative- CPB-CC-PDS, the CPB-CC-PDS-infected plants were moved into Adaptis A1000 growth chambers (Conviron, Winnipeg, Manitoba,Canada),with temperature set at 18 °C. Inoculated leaves (IL) samples were collected at 5 days post inoculation(dpi) while upper young leaves (UL) were collected at 10 dpi for analysis. Leaves from six different plants, one leaf per plant, were pooled before RNA or protein extractions to minimize the sampling errors.

### RNA blot analysis

Total RNAs were extracted from infected plants and subjected to RNA blot analysis to detect TCV viral RNAs, or vsRNAs using protocols outlined in previous reports^2^. 0.8 μg of Arabidopsis RNA were used to detect viral RNA, while for vsRNAs detection 10 μg of Arabidopsis RNA were used. The probes were a mixture of five different oligonucleotides end labeled with ^32^P.

## Additional Information

**How to cite this article**: Zhang, X. *et al*. Incomplete DRB4-dependence of the DCL4-mediated antiviral defense. *Sci. Rep.* **6**, 39244; doi: 10.1038/srep39244 (2016).

**Publisher's note:** Springer Nature remains neutral with regard to jurisdictional claims in published maps and institutional affiliations.
